# Impact of type 2 diabetes treated with non-insulin medication and number of diabetes-coexisting diseases on EQ-5D-5 L index scores in the Finnish population

**DOI:** 10.1186/s12955-019-1187-9

**Published:** 2019-07-08

**Authors:** Kari Jalkanen, Emma Aarnio, Piia Lavikainen, Hanna-Mari Jauhonen, Hannes Enlund, Janne Martikainen

**Affiliations:** 1 School of Pharmacy, University of Eastern Finland, P.O. Box 1627, 70211 Kuopio, Finland; 2 School of Pharmacy, University of Eastern Finland, Finland Institute of Biomedicine, University of Turku, Finland University of Eastern Finland, P.O. Box 1627, 70211 Kuopio, Finland; 3 Finnish Medicines Agency, FIMEA, P.O. Box 55, 00034 Kuopio, Finland

**Keywords:** EQ-5D-5 L, Type 2 diabetes mellitus, Health-related quality of life, Quality-adjusted life year

## Abstract

**Background:**

Type 2 diabetes (T2D) causes significant health and economic burden. In addition to comorbidities there are also coexisting diseases linked to obesity, lifestyle and T2D. The aim of this study was to examine the effect of T2D and T2D-coexisting diseases on health-related quality of life (HRQoL) in the Finnish population and whether it is T2D or the coexisting diseases that have the largest impact on HRQoL.

**Methods:**

The study was based on a national cross-sectional population survey (*n* = 5305). Respondents’ HRQoL was measured using the EQ-5D-5 L instrument. Our study included diabetic respondents treated with non-insulin medications (NI-T2D) with or without insulin and non-diabetic respondents, whereas diabetic respondents not taking any anti-diabetic medications or treated with insulin alone were excluded. A crosswalk algorithm was used to convert EQ-5D-5 L index scores into EQ-5D-3 L index scores as a sensitivity analysis. A two-part model was used to examine the association between T2D and coexisting diseases and HRQoL.

**Results:**

The unadjusted mean (SD) EQ-5D-5 L index scores for non-diabetics (*n* = 4856) was 0.90 (0.13) and 0.85 (0.16) for respondents with NI-T2D (*n* = 449). With adjustment for demographic factors, the difference in EQ-5D-5 L index scores was 0.036 (95% CI 0.023–0.050). After adjusting for the number of coexisting diseases, the EQ-5D-5 L index scores among respondents with NI-T2D and three or more coexisting diseases were lower when compared to all non-diabetics but not when compared to non-diabetics with similar number of coexisting diseases. The number of T2D-coexisting diseases had a larger effect on EQ-5D-5 L index scores in younger age groups (20 and 40 years old).

**Conclusions:**

Lower EQ-5D-5 L index score is associated with NI-T2D when compared to non-diabetic respondents. When compared to non-diabetics, the disutility associated with NI-T2D increases as more coexisting diseases appear. The disutility effect of coexisting diseases was equally large in non-diabetics and respondents with NI-T2D. Thus, public health interventions targeting the prevention of both T2D and its coexisting diseases have potential to have significant benefits also in terms of HRQoL.

**Electronic supplementary material:**

The online version of this article (10.1186/s12955-019-1187-9) contains supplementary material, which is available to authorized users.

## Background

The prevalence of lifestyle-related non-communicable diseases is increasing rapidly in many high-income countries; including Finland [1]. Increasing prevalence of overweight and obesity, arising from energy-rich diets and sedentary lifestyles, is driving this growth [2]. Overweight and obesity are significant risk factors for T2D [3] and many other prevalent diseases, including hypertension, cancer, arthritis, obstructive sleep apnea, and gall bladder disease [4]. Thus, there is an interest to examine the connections between obesity, type 2 diabetes (T2D) and its coexisting diseases.

Globally, diabetes causes a considerable disease burden with an estimated 425 million adults having diabetes in 2017, also carrying along a major economic burden [5]. For example, in Finland, the amount of people with T2D is estimated to be 500,000 and the number has been growing every year at an average rate of 4.2% during the last eight years [6, 7]. The estimated cost of T2D was 1.5 billion euros in Finland in 2011 [8].

Decision makers in healthcare require information on the health-related quality of life (HRQoL) impact of different diseases. HRQoL is a part of a broader quality of life (QoL) concept that focuses more on physical or mental aspects of a person’s welfare and the person’s functional ability [9]. This allows more accurate measurement of the effects of, for example, preventive interventions via the use of patient reported outcomes. In a Finnish study, T2D has been shown to be preventable via lifestyle interventions [10]. Through the prevention of T2D it is possible to also better conserve HRQoL on the population level.

Recently, studies have been performed on the HRQoL in T2D patients and the effect of coexisting diseases using the EQ-5D-5 L instrument [11–15]. However, to our knowledge, studies where persons with T2D have been compared with non-diabetics using the EQ-5D-5 L instrument have not yet been done. The aim of the present study was to examine the difference in HRQoL between non-diabetic respondents and respondents with T2D in a relatively large, nationally representative population sample by using the EQ-5D-5 L instrument [16] as well as to examine whether it is T2D or the coexisting diseases that have the largest impact on HRQoL. The diseases we selected for this study were those that are known to coexist with obesity, overweight, and T2D: elevated blood cholesterol, hypertension, kidney problems, musculoskeletal disorders (i.e., joint pain, nerve pain), gastrointestinal problems, heart disease and sleeping problems [17–20].

## Methods

### Data source and study population

The Medicines Barometer carried out by the Finnish Medicines Agency (Fimea) is designed to study opinions, values and experiences related to medicines, health and well-being of the Finnish population. Barometer carried out in 2015 was a cross-sectional survey using two different data collection methods: a postal survey and an internet survey [21]. The survey was executed by a market research company (Taloustutkimus Ltd.). Respondents were randomly selected from the national population register and stratified by gender, age, and residential area for the postal survey, which was sent to 8003 persons aged 18–80. The internet survey was sent to 13,900 persons stratified by gender, age and area of residence from an internet panel of Taloustutkimus Ltd. The Medicines Barometer consisted of a series of demographic questions, questions about use of medications, illnesses and the EQ-5D-5 L instrument. The EQ-5D instrument has been shown to be a valid measure of HRQoL in T2D [22, 23]. HRQoL was measured using the Finnish translation of the EQ-5D-5 L instrument on the EuroQol Research Foundation server. In order to increase the validity of our study, the EQ-5D crosswalk scores for conversion into EQ-5D-3 L index scores were incorporated into our results as is recommended by the EuroQol foundation [24].

**Fig. 1:**
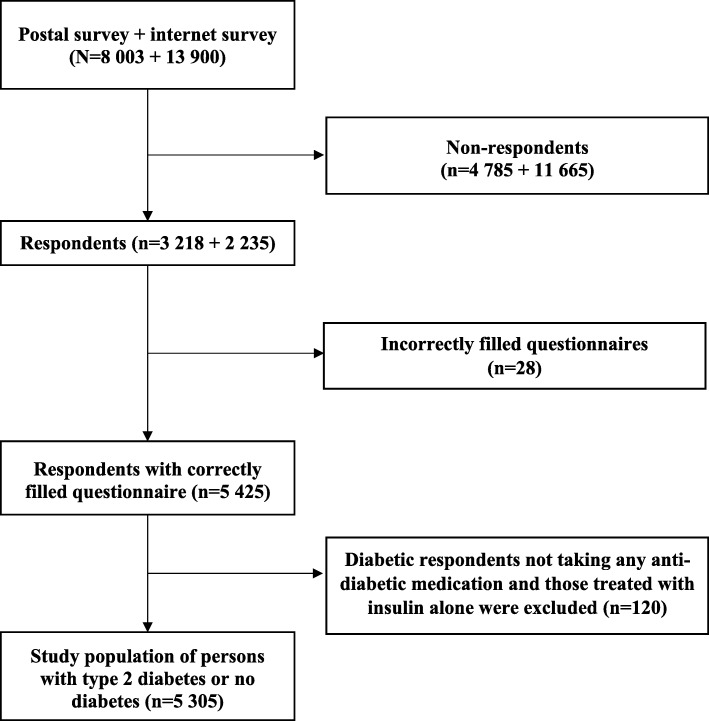
Flow diagram depicting the respondents in the study

Respondents with diabetes were identified from the Medicines Barometer according to their answer to the question “Do you have diabetes”. Furthermore, respondents with non-insulin treated T2D (NI-T2D) were identified as those who reported use of other diabetes medication besides insulin within the last seven days or reported to have a healthcare reimbursement number for other diabetes medication besides insulin. In the Finnish healthcare setting, patients may have a reimbursement number that is associated either with a specific medication (e.g., liraglutide) or with a specific disease such as asthma [25]. Patients who reported to have used both insulin and non-insulin medication were also classified as NI-T2D patients. Diabetic respondents not taking any anti-diabetic medications or treated with insulin alone (i.e., no use of any non-insulin medications) were excluded. A flow diagram of the study population is presented in Fig. 1.

### Ethical statement

The study setting and research process complied with local and national ethical instructions for research [12]. All respondents received a survey cover letter containing an explanation of the purpose of the study and the handling of the obtained data. In accordance with the national ethical instructions for research [12], answering the survey was regarded as an informed consent to participate in the survey. Thus, no ethical board approval was required for this study. Furthermore, only the market research company Taloustutkimus Ltd. knew the identity of the respondents and destroyed the records two months after data collection was finished.

### EQ-5D

The EQ-5D-5 L instrument is used to measure HRQoL. It consists of five dimensions and five levels forming a matrix of 3125 possible health states. The purpose of EQ-5D-5 L instrument is to better measure changes in health near the best health state (i.e., 11,111) than the EQ-5D-3 L instrument [26]. The EQ-5D-5 L instrument provides an index score between 1 and − 0.285 depicting the HRQoL state [27]. The interpretation of the score is so that an index score of 1 means perfect health, 0 means death and index scores below 0 mean states worse than death. At the moment, EQ-5D-5 L value set for UK has not yet been validated [28].

### Covariates

The EQ-5D-5 L index scores were adjusted according to the Organization for Economic Cooperation and Development (OECD) social indicators [29]. These were a part of the original questionnaire and included marital status, highest level of education, employment, age, sex, and income. Adjustments were also made for the survey type (internet or postal).

We selected comorbidities which are known to coexist with obesity, overweight, and T2D. Other identified diseases or conditions were referred to as “other diseases”. Coexisting diseases were identified from the questionnaire using a multi-state sorting procedure: Persons could select a disease from a provided list of diseases or provide a free text answer. In addition, they were asked to provide their medical reimbursement codes, which in Finland, are associated with a specific disease (e.g., number 205 refers to hypertension) or they may be associated with a specific medicine in a specific indication (e.g., 171 for insulin degludec). Lastly, they were asked about medications they had used during the last week. All the answers were sorted by the researchers and if any one of these three questions (i.e., diseases, reimbursement codes, used medication) were linked to a disease, the person was categorized as having that disease. The identification was verified by a clinician expert.

### Statistical analysis

Demographic characteristics between respondents with NI-T2D and non-diabetic respondents were compared using Chi Square tests for categorical variables and independent samples t-tests for continuous ones. Index score is a cardinal value that represents the strength of a person’s preference to a specific health state. A two-part model was used to examine the association between T2D and disutility score (i.e. 1 – EQ-5D-5 L index score) due to the skewness of the index score data (i.e., high frequency of persons with perfect health). The two-part models consisted of a logistic regression as a first part for the probability of disutility and a generalized linear model with gamma distribution and log link function as a second part for the average disutility score conditional on disutility. For the second part, models with gamma distribution and identity link function were also examined, but a model with log link function was selected as the best fitting based on comparisons of Akaike Information Criteria between the models. Analyses were adjusted for OECD social indicators (age, income, education, employment, residential area and marital status), number of T2D-coexisting (categorized as 0, 1, 2, 3, and ≥ 4) and all other diseases and the survey type (postal or internet). Marginal effects of NI-T2D as well as other covariates were estimated for the entire sample. Sensitivity analysis was carried out by performing a crosswalk algorithm with the Crosswalk Index Value Calculator provided by EuroQoL [24]. This transformed the value set of EQ-5D-5 L into the validated EQ-5D-3 L version. Subgroup analyses were also conducted for respondents of one single age: 20, 40, 60 and 80 years old. Data were analyzed using IBM SPSS Statistics (IBM Corp, New York, USA) version 25 and STATA/SE (StataCorp, Texas, USA) version 14.2. Figures were drawn using R Studio version 3.3.4 (The R Foundation for statistical computing, Vienna, Austria). Two-part models were estimated with STATAs twopm command and marginal effects with margins command. Results were considered statistically significant for *p* < .05 in all analyses.

## Results

In total, there were 5425 survey respondents. The size of the sample used in this study was 5305 after diabetic respondents not taking any anti-diabetic medications or treated with insulin alone (*n* = 120) were excluded (*n* = 120). Number of male respondents was 2349 (44.4%) and mean (SD) age of the respondents was 53.4 (16.2) years [Table 1]. Of the 5305 respondents, 449 were identified as having NI-T2D, and 4856 were categorized as having no diabetes.

**Table 1:** Demographics of respondents

		Population (*n* = 5305) n (%)	Non-insulin treated type 2 diabetes (*n* = 449) n (%)	No diabetes (*n* = 4856) n (%)
	Male*	2349 (44.4)	248 (55.4)	2101 (43.3)
Age (year)*				
	Mean ± SD	53.4 ± 16.2	65.0 ± 9.4	52.4 ± 16.3
	Range	18–80	21–80	18–80
Questionnaire type	Internet survey*	2196 (41.4)	160 (35.6)	2036 (41.9)
Diabetes-coexisting diseases	Hypertension*	1564 (29.5)	349 (77.7)	1215 (25.0)
	Hypercholesterolemia *	1078 (20.3)	298 (66.4)	780 (16.1)
	Musculoskeletal disorders*	838 (15.8)	116 (25.8)	722 (14.9)
	Heart problems*	581 (11.0)	134 (29.8)	447 (9.2)
	Sleep problems*	383 (7.2)	68 (15.1)	315 (6.5)
	GI disorders*	330 (6.2)	45 (10.0)	285 (5.9)
	Kidney disease	10 (0.2)	2 (0.4)	8 (0.2)
Number of diabetes-coexisting diseases*	0	2682 (50.5)	30 (6.6)	2652 (54.6)
	1	1281 (24.1)	97 (21.6)	1184 (24.3)
	2	734 (13.8)	137 (30.5)	597 (12.2)
	3	434 (8.1)	122 (27.2)	312 (6.3)
	4 or more	174 (3.2)	63 (14.0)	111 (2.2)
Marital status*	Married	3197 (60.4)	247 (61.4)	2923 (60.4)
	Unmarried	1107 (20.9)	56 (12.6)	1051 (21.7)
	Divorced or separated	643 (12.2)	57 (12.8)	586 (12.1)
	Widowed	342 (6.5)	59 (13.2)	283 (5.8)
Level of education*	Elementary school	779 (14.8)	126 (28.2)	653 (13.5)
	High school	415 (7.9)	16 (3.6)	399 (8.3)
	Vocational school	1979 (37.6)	182 (40.7)	1797 (37.3)
	College	851 (16.2)	52 (11.6)	799 (16.6)
	University	1120 (21.3)	55 (12.3)	1065 (22.1)
	Other	124 (2.4)	16 (3.6)	108 (2.2)
Household income*	Less than 1000 €	538 (10.3)	48 (10.9)	490 (10.3)
	1001–2000 €	1292 (24.8)	134 (30.5)	1158 (24.3)
	2001–3000 €	1219 (23.4)	115 (26.1)	1104 (23.1)
	3001–4000 €	1053 (20.2)	79 (18.0)	974 (20.4)
	4001–5000 €	619 (11.9)	36 (8.2)	583 (12.2)
	5001–8000 €	403 (7.7)	21 (4.8)	382 (8.0)
	Over 8000 €	91 (1.8)	7 (1.6)	84 (1.8)
Employment*	Full time work	2094 (39.6)	74 (16.5)	2020 (41.8)
	Part time work or retirement	346 (6.6)	23 (5.1)	323 (6.7)
	Unemployed or laid off	374 (7.1)	26 (5.8)	348 (7.2)
	Retired	1942 (36.8)	313 (69.9)	1629 (33.7)
	Other	526 (10.0)	12 (2.7)	415 (10.6)

*p < .05 when comparing respondents with non-insulin treated type 2 diabetes to respondents with no diabetes. *GI* Gastrointestinal, *SD* Standard deviation

Non-diabetic respondents reported slightly better scores in the mobility, usual activities and pain/discomfort dimensions of the EQ-5D-5 L questionnaire [Additional file 1]. The difference was most clearly visible in the pain/discomfort dimension. Results from the anxiety/depression and self-care dimensions did not differ between respondents with NI-T2D and non-diabetics. When comparing frequency of respondents with a score of 1 (“No problems”) to those with any other score, there was a statistically significant difference between respondents with NI-T2D and non-diabetics in all other categories besides anxiety/depression.

There were differences between the five most frequently reported health states between non-diabetics and respondents with NI-T2D [Additional file 2]. The most frequently reported health state for non-diabetics was 11,111 (29.3%) whereas 11,121 (17.4%) was the most frequently reported health state among those with NI-T2D with a worse score in the pain/discomfort dimension.

Prevalence of the T2D-coexisting diseases (as defined earlier) was greater among respondents with NI-T2D compared to non-diabetics [Table 1]. For example, hypertension was more prevalent among respondents with NI-T2D (77.7% vs 25.0%). Hypercholesterolemia was more prevalent among respondents with NI-T2D (66.4% vs 16.1%). Of the T2D-coexisting diseases, musculoskeletal disorder (i.e., joint pain) had the largest impact on EQ-5D-5 L index scores, 0.097 (SD 0.005) as measured with the EQ-5D-5 L index score. The postal survey respondents were older than internet survey respondents and had a lower EQ-5D-5 L index score [Additional file 3]. NI-T2D was more common among postal survey respondents (9.3%) compared to internet survey respondents (7.3%).

**Fig. 2:**
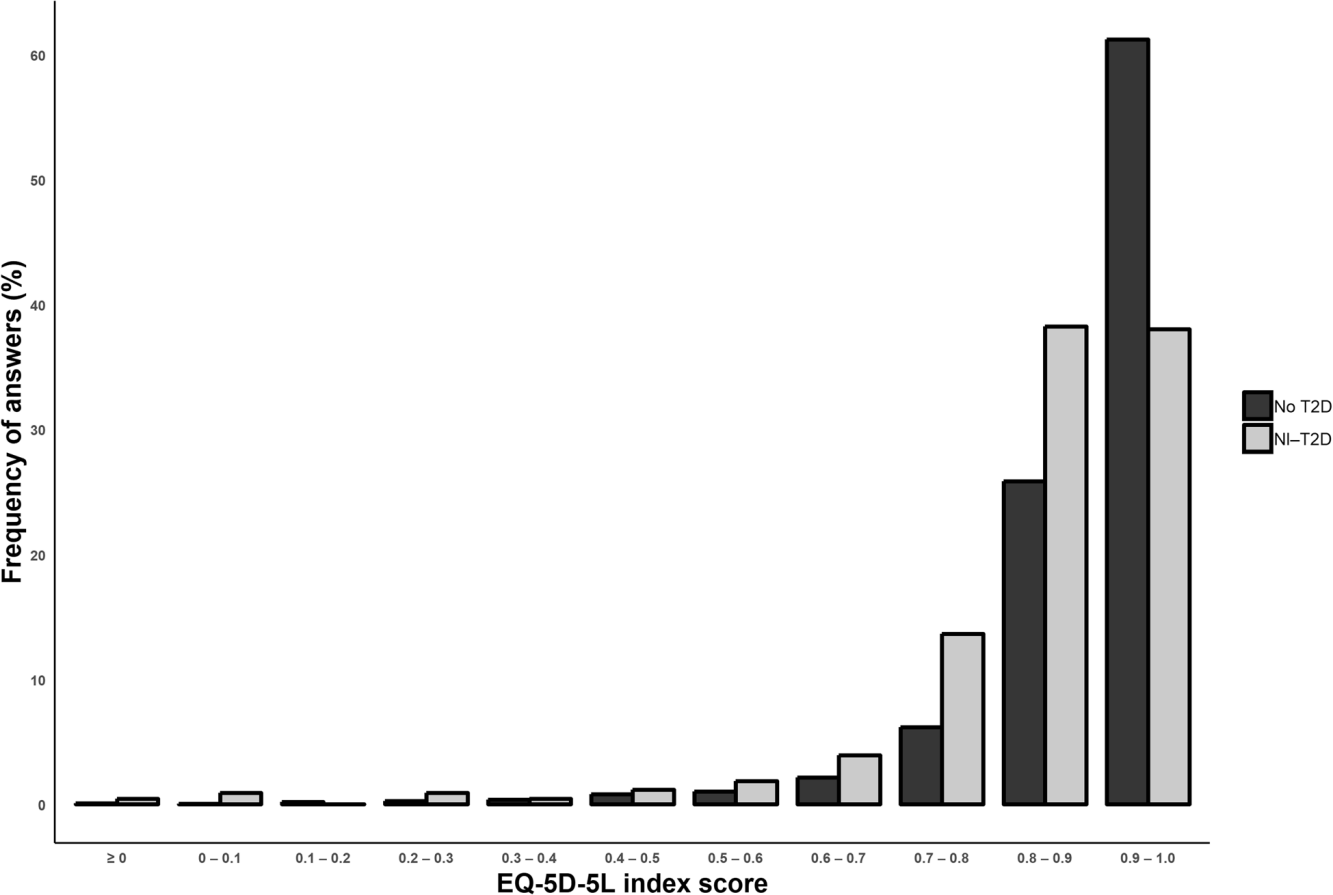
Frequencies of EQ-5D-5 L index scores

Mean (SD) EQ-5D-5 L index score was 0.90 (0.77–1.03) among respondents without diabetes and 0.85 (0.69–1.01) among respondents with NI-T2D [Fig. 2]. Difference between the groups was statistically significant (*p* < .001). The unadjusted mean (SD) crosswalk EQ-5D-3 L index scores were 0.83 (0.68–0.98) for non-diabetic respondents and 0.76 (0.58–0.94) for respondents with NI-T2D.

After adjusting for sociodemographic factors, the EQ-5D-5 L index scores among respondents with NI-T2D were 0.036 points (95% CI 0.023–0.050) lower than among non-diabetics [Additional file 4A] and 0.047 (95% CI 0.030–0.062) lower when using the crosswalk EQ-5D-3 L index scores [Additional file 4B]. When the results were adjusted for the presence of all diseases and sociodemographic factors, the difference in the scores was no longer statistically significant (0.010, 95% CI − 0.001 to 0.021).

Of respondents with NI-T2D, 41.2% had at least three coexisting diseases [Table 2]. When comparing the EQ-5D-5 L index scores of respondents with NI-T2D with different number of T2D-coexisting diseases to all non-diabetics (i.e., having any number of T2D-coexisting diseases) in a two-part model, the HRQoL declining effect to HRQoL became statistically significant when the number of T2D-coexisting diseases exceeded three [Table 3]. The number of coexisting diseases was a related to decrease in EQ-5D-5 L index scores but there was no difference between non-diabetics and respondents with NI-T2D with same number of coexisting diseases.

**Table 2:** The prevalence of T2D-coexisting diseases in respondents with NI-T2D and non-diabetics classified by their amount (n)

Number of coexisting diseases (n)	Hypertension n (%)	Hypercholesterolemia n (%)	Musculoskeletal disorders n (%)	Heart problems n (%)	Sleep problems n (%)	GI disorders n (%)	Kidney disease n (%)
NI-T2D							
1 (97)	61 (62.9)	24 (24.7)	3 (3.1)	2 (2.1)	4 (4.1)	3 (3.1)	0 (0.0)
2 (137)	115 (83.9)	106 (77.4)	16 (11.7)	18 (13.1)	12 (8.8)	7 (5.1)	0 (0.0)
3 (122)	111 (91.0)	108 (88.5)	47 (38.5)	66 (54.1)	20 (16.4)	13 (10.7)	1 (0.8)
≥ 4 (63)	62 (98.4)	60 (95.2)	50 (79.4)	48 (76.2)	32 (50.8)	22 (34.9)	1 (1.6)
Non-diabetics							
1 (1184)	448 (37.8)	160 (13.5)	288 (24.3)	81 (6.8)	95 (8.0)	112 (9.5)	0 (0.0)
2 (597)	390 (65.3)	291 (48.7)	212 (35.5)	112 (18.8)	106 (17.8)	81 (13.6)	2 (0.3)
3 (312)	269 (86.2)	233 (74.7)	139 (44.6)	175 (56.1)	62 (19.9)	53 (17.0)	5 (1.6)
≥ 4 (111)	108 (97.3)	96 (86.5)	83 (74.8)	79 (71.2)	52 (46.8)	39 (35.1)	1 (0.9)

*GI* Gastrointestinal

**Table 3:** Marginal effects of NI-T2D and coexisting diseases on the HRQoL of respondents as measured with the EQ-5D-5 L and EQ-5D-3 L crosswalk values

Non-diabetics without coexisting diseases as reference group	Difference in quality of life compared to non-diabetic persons with no coexisting diseases	
Category, number of coexisting diseases	EQ-5D-5 L index score (95% CI)	EQ-5D-3 L crosswalk index score (95% CI)
No T2D, 0	0 (reference)	0 (reference)
No T2D, 1	−0.053 (−0.061 to −0.046)	−0.079 (−0.089 to −0.069)
No T2D, 2	−0.091 (− 0.103 to − 0.079)	− 0.121 (− 0.135 to − 0.107)
No T2D, 3	− 0.100 (− 0.116 to − 0.083)	−0.136 (− 0.155 to − 0.118)
No T2D, 4 ≥	−0.196 (− 0.235 to − 0.157)	−0.227 (− 0.261 to − 0.193)
NI-T2D, 0	−0.015 (− 0.051 to 0.020)	−0.021 (− 0.073 to 0.031)
NI-T2D, 1	− 0.060 (− 0.084 to − 0.036)	−0.083 (− 0.113 to − 0.053)
NI-T2D, 2	−0.063 (− 0.084 to − 0.041)	−0.086 (− 0.112 to − 0.060)
NI-T2D, 3	−0.097 (− 0.122 to − 0.073)	−0.133 (− 0.160 to − 0.106)
NI-T2D, 4 ≥	−0.222 (− 0.279 to − 0.164)	−0.247 (− 0.296 to − 0.199)
All non-diabetics together as reference group	Difference in quality of life compared to non-diabetic persons	
Number of coexisting diseases	EQ-5D-5 L index score (95% CI)	EQ-5D-3 L crosswalk index score (95% CI)
No T2D	0 (reference)	0 (reference)
NI-T2D, 0	0.028 (−0.006 to 0.062)	0.039 (−0.012 to 0.090)
NI-T2D, 1	−0.014 (− 0.038 to 0.009)	−0.020 (− 0.049 to 0.010)
NI-T2D, 2	− 0.013 (− 0.033 to 0.007)	−0.019 (− 0.044 to 0.007)
NI-T2D, 3	− 0.043 (− 0.066 to − 0.019)	−0.064 (− 0.091 to − 0.036)
NI-T2D, ≥4	−0.152 (− 0.207 to − 0.097)	−0.170 (− 0.219 to − 0.121)

Results were adjusted for age, sex, income, education, marital status, survey type and employment

**Fig. 3:**
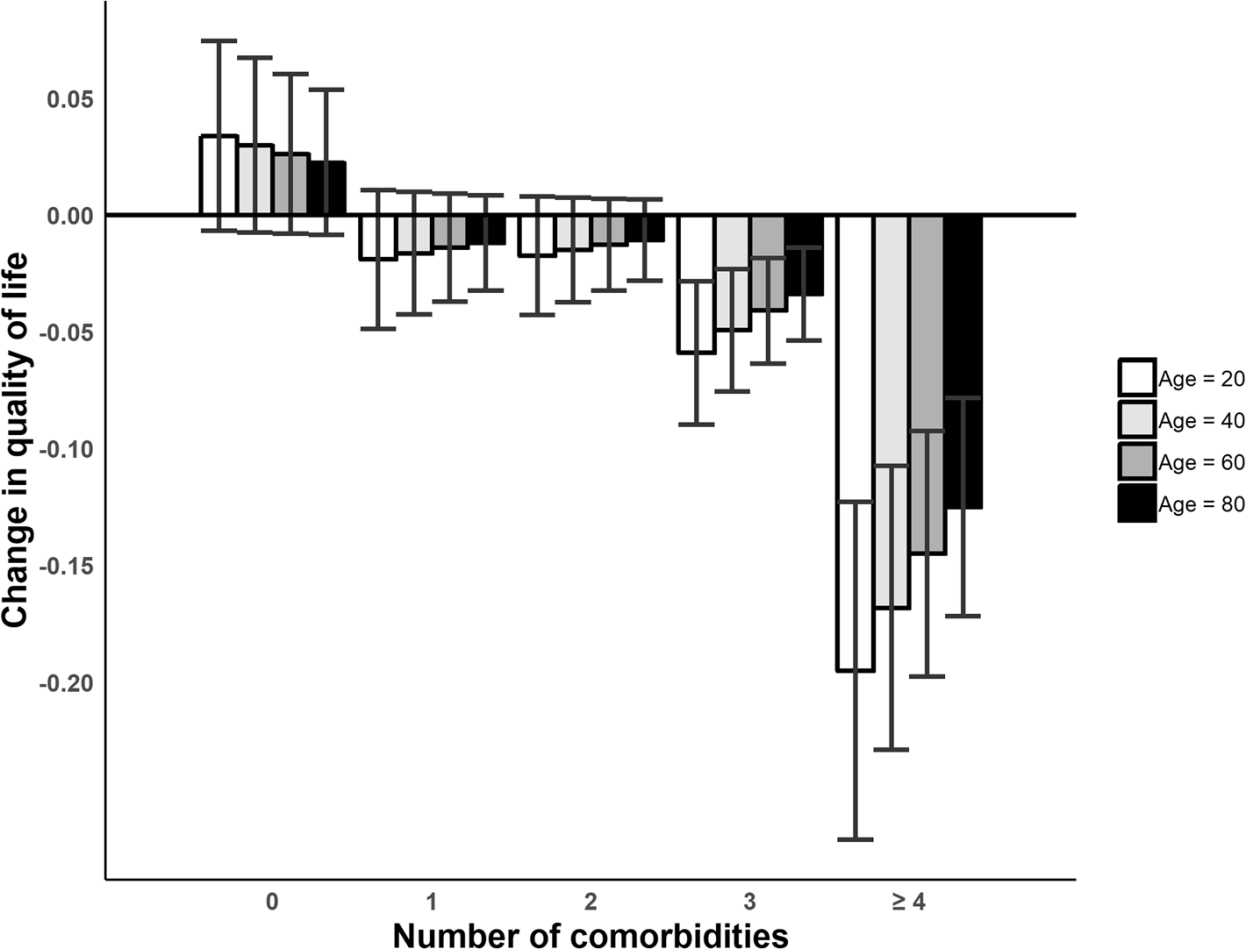
Marginal effects of coexisting diseases on the HRQoL of respondents with NI-T2D as measured with the EQ-5D-5 L values according to four different age groups (20, 40, 60 and 80) compared with non-diabetic persons. Results were adjusted for sex, income, education, marital status, survey type and employment

In the subgroup analyses, lower utility values were more frequent in younger respondents (20 and 40 years old) with NI-T2D when compared with persons without diabetes [Fig. 3]. Three or more T2D-coexisting diseases lead to a statistically significant difference in HRQoL when compared to non-diabetic persons. When compared to non-diabetics with no coexisting diseases by the marginal effect point estimates, there was no difference in disutility scores between non-diabetics and respondents with NI-T2D in single ages 20, 40, 60 and 80 years old. These results are shown in Additional file 5.

## Discussion

Based on the present study, NI-T2D is associated with lower EQ-5D-5 L index scores compared to non-diabetics but only if T2D-coexisting diseases are present. For respondents with NI-T2D, the mean HRQoL was lower than for non-diabetics when measured with both EQ-5D-5 L and crosswalk EQ-5D-3 L index scores. This difference did not disappear after adjusting for sociodemographic factors. When looking at the effect of T2D-coexisting diseases on the HRQoL, respondents with three or more T2D-coexisting diseases in addition to NI-T2D had a statistically significant decrease in HRQoL and this decrease became even larger when the EQ-5D-crosswalk algorithm was used. The most significant decrease in the EQ-5D-5 L index scores was observed among younger age groups. The difference between non-diabetics and respondents with NI-T2D disappeared when the results were adjusted for all present diseases. There was also no difference in EQ-5D-5 L index scores between non-diabetics and respondents with NI-T2D that had the same number of T2D-coexisting diseases. Hypertension and hypercholesterolemia were the most common coexisting diseases in persons with NI-T2D followed by heart problems and musculoskeletal disorders.

Our results on the effect of diabetes on HRQoL are similar to most previous studies using EQ-5D-3 L [30, 31], EQ-5D-3 L and SF-6D [32] and EQ-5D-5 L [12, 33]. In a previous Finnish study, diabetes was found to reduce HRQoL by 0.041 (SD 0.012) when measured with the EQ-5D-3 L instrument [34]. A recent study conducted in Canada determined the minimally important difference (MID) for T2D to be between 0.03 and 0.05 when measured with the EQ-5D-5 L [35]. Using the standard EQ-5D-5 L index scores and crosswalk EQ-5D-3 L index scores, the differences in our study were 0.036 and 0.047, respectively.

Our results are also in line with previous studies where T2D and/or type 1 diabetes and the presence of comorbidities among T2D patients have been connected to a lower HRQoL [11, 36–39]. For example, the decrease in HRQoL became statistically significant when the number of comorbid conditions exceeded three in a previous study among the Dutch population indicating that identifying those patients with disease burdens is crucial [11].

Studies covering multiple diseases have shown a slight negative effect of diabetes on HRQoL [12, 34, 40]. Our study supports the result where a type of dose-response relationship has been observed between HRQoL and the number of comorbidities [11, 12]. In the present study, the HRQoL reducing effect of NI-T2D disappeared after adjusting for all other diseases. It is therefore possible that other diseases themselves play a larger role in the HRQoL experienced by patients rather than just the diagnosis of T2D, and according to our results, this role may be larger especially in younger age groups.

Our findings together with previous studies suggest that a significant improvement in population’s HRQoL could be reached by preventing T2D with lifestyle interventions [14, 41]. In addition, musculoskeletal disorders [42], gastrointestinal disorders [4, 43], sleep problems [18], hypertension, and hypercholesterolemia [4] are connected to obesity and thus also metabolic disorder and T2D. Improvement in HRQoL could thus be achieved by, for example, targeting the population via campaigns and public health programs [44]. If both T2D and its coexisting diseases can be targeted simultaneously, a greater HRQoL benefit can be achieved.

The strengths of our study are that the sample size in our study was relatively large (*n* = 5305) and the Medicines Barometer -survey is planned to be repeated bi-annually by Fimea offering a possibility for further studies. Second, we were able to analyze the results using both EQ-5D-5 L and crosswalk EQ-5D-3 L index scores to better understand the underlying differences between these two value sets as the discussion on the validity of the EQ-5D-5 L instrument is ongoing [45, 46]. When writing this article, NICE released a statement concluding that the EQ-5D-3 L-instrument is to be used for reference case analyses and that the EQ-5D-5 L-instrument still needs more data to support its validity [47]. Third, in this study the lower index scores that were produced by the EQ-5D-5 L index score compared to the crosswalk EQ-5D-3 L index scores are in line with a theoretical analysis done in a previous study [28].

There are some limitations that should be noted in this study. As is common for QoL data, the HRQoL results are skewed in a way that higher scores were reported more than lower scores [48]. A ceiling effect was seen in the results. This means our measurements were only able to partially capture the variation in health states among respondents. Also, the population was demographically different between the internet survey and the postal survey respondents, and the response rate was lower for the internet survey (16.0% vs 40.2%) [21].

In addition, this study was also not able to identify all respondents with T2D since the answers were not complete (i.e., a person could state that they use a medicine for treating diabetes, but they did not specify if they used only insulin or not). In our study, we were not able to measure the effects of blood glucose on the HRQoL of the respondents. In another study, this was found to have a minor decreasing effect on HRQoL [12]. The choice of coexisting diseases that are related to T2D could also affect the results. We chose to only focus on those that are coexisting with T2D and, therefore, these could be overrepresented in the T2D population since, for example, the treatment criteria for hypercholesterolemia and hypertension are different between non-diabetics and T2D population. The categorization of the T2D-coexisting diseases was limited by the structure of the questionnaire. For example, it was not possible to differentiate between different kinds of heart related conditions since the question on the form was “Do you have a heart disease – yes or no”. Furthermore, when making this study, a Finnish value set for EQ-5D-5 L was not currently available. For this, we had to resort to using a UK value set instead. This value set was chosen because previous research has shown a similarity between the UK and the Finnish value set [34]. This may possibly affect the results of this study since cultural differences affect the EQ-5D questionnaire results [49, 50]. The development of a Finnish value set would increase the validity of results in HRQoL studies conducted in the Finnish population.

## Conclusions

Both unadjusted and adjusted EQ-5D-5 L index scores were lower among respondents with NI-T2D compared to non-diabetics. When adjusted for the number of coexisting diseases, the EQ-5D-5 L index scores of respondents with NI-T2D were lower compared to non-diabetics if respondents with NI-T2D had three or more T2D-coexisting diseases present. This effect was more visible in the younger respondents.

## Additional files

Additional file 1: Comparison of EQ-5D-5 L levels between non-diabetics and respondents with NI-T2D. EQ-5D-5 L dimensions of non-diabetics and respondents with NI-T2D listed by their appearing frequencies. (DOCX 15 kb)

Additional file 2: Five most frequently reported EQ-5D-5 L health states in persons with NI-T2D and non-diabetics. Five most frequently reported EQ-5D-5 L health states in respondents with NI-T2D and non-diabetics. The dimensions are in order: mobility, self-care, usual activities, pain/discomfort and anxiety/depression. (DOCX 14 kb)

Additional file 3: Characteristics of respondents who attended the postal survey and the internet survey. An overview of the demographics and the disease burden of the postal survey respondents and internet survey respondents. (DOCX 17 kb)

Additional file 4: A Marginal effects estimated with two-part model for the association between NI-T2D and EQ-5D-5 L disutility score (i.e., 1 – EQ-5D-5 L index score) (*N* = 4998). The marginal effects of the two-part model used in calculating EQ-5D-5 L index scores. 4 B Marginal effects estimated with two-part model for the association between NI-T2D and crosswalk EQ-5D-3 L disutility score (i.e., 1 – crosswalk EQ-5D-3 L index score) (*N* = 4998). The marginal effects of the two-part model used in calculating EQ-5D-5 L/3 L Crosswalk index scores. (ZIP 34 kb)

Additional file 5: EQ-5D-5 L disutility scores of respondents with NI-T2D when compared to non-diabetics with no coexisting diseases. The observed differences are the marginal effect point estimates and their 95% confidence intervals. EQ-5D-5 L disutility scores of respondents with NI-T2D when compared to non-diabetics with no coexisting diseases. Comparison done in respondents of a single age: 20, 40, 60 and 80 years and accumulated coexisting diseases (0, 1, 2, 3 or 4 ≥). (DOCX 18 kb)

## Data Availability

All data generated or analyzed during this study are included in this published article [and its supplementary information files].

## Abbreviations

CI: Confidence interval
EQ-5D-3 L: EuroQol – 3 dimensions
EQ-5D-5 L: EuroQol – 5 dimensions
Fimea: Finnish Medicines Agency
HRQoL: Health-related quality of life
MID: Minimally important difference
NI-T2D: Non-insulin treated type 2 diabetes
OECD: Organization for Economic Cooperation and Development
QoL: Quality of Life
SD: Standard deviation
T2D: Type 2 diabetes
