# Supplementary material for: Impact of type 2 diabetes treated with non-insulin medication and number of diabetes-coexisting diseases on EQ-5D-5 L index scores in the Finnish population
Source: Health Qual Life Outcomes. 2019 Jul 8;17:117. doi: 10.1186/s12955-019-1187-9 (PMC6615142; doi:10.1186/s12955-019-1187-9)
Supplement: Supplementary file 1 — Comparison of EQ-5D-5 L levels between non-diabetics and respondents with NI-T2D. EQ-5D-5 L dimensions of non-diabetics and respondents with NI-T2D listed by their appearing frequencies. (DOCX 15 kb) [file 12955_2019_1187_MOESM1_ESM.docx]

Additional file 1. Comparison of EQ-5D-5L levels between non-diabetics and respondents with NI-T2D.

| EQ-5D-5L dimensions | **No diabetes**  **n (%)** | | |  | |  | | **Total** | **NI-T2D**  **n (%)** | | |  | |  | | **Total** |
| --- | --- | --- | --- | --- | --- | --- | --- | --- | --- | --- | --- | --- | --- | --- | --- | --- |
|  | 1 | 2 | 3 | | 4 | | 5 |  | 1 | 2 | 3 | | 4 | | 5 |  |
| Mobility | 3 619  (75.8 %) | 825  (17.2 %) | 229  (4.7 %) | | 88  (1.8 %) | | 10  (0.2 %) | 4 771 | 194  (43.8 %) | 173  (39.1 %) | 49  (11 %) | | 25  (5.6 %) | | 1  (0.2 %) | 442 |
| Self-care | 4 545  (95.3 %) | 161  (3.3 %) | 46  (0.9 %) | | 13  (0.2 %) | | 4  (0.1 %) | 4 769 | 403  (91.3 %) | 24  (5.4 %) | 10  (2.2 %) | | 3  (0.6 %) | | 1  (0.2 %) | 441 |
| Usual activities | 3 839  (80.6 %) | 634  (13.3 %) | 195  (4.0 %) | | 66  (1.3 %) | | 27  (0.5 %) | 4 761 | 293  (67.2 %) | 91  (20.8 %) | 33  (7.5 %) | | 17  (3.8 %) | | 2  (0.4 %) | 436 |
| Pain/Discomfort | 1 667  (34.9 %) | 2 408  (50.4 %) | 606  (12.6%) | | 85  (1.7 %) | | 8  (0.1 %) | 4 774 | 87  (19.6 %) | 220  (49.7 %) | 117  (26.4 %) | | 15  (3.3 %) | | 3  (0.6 %) | 442 |
| Anxiety/Depression | 3 558  (74.6 %) | 990  (20.7 %) | 165  (3.4 %) | | 38  (0.7 %) | | 13  (0.2 %) | 4 764 | 311  (71.1 %) | 105  (24 %) | 14  (3.2 %) | | 5  (1.1 %) | | 2  (0.4 %) | 437 |

EQ-5D-5L levels:

1 – No problems, 2 – Slight problems, 3 – Moderate problems, 4 – Severe problems, 5 – Unable to perform actions
