# Supplementary material for: Impact of type 2 diabetes treated with non-insulin medication and number of diabetes-coexisting diseases on EQ-5D-5 L index scores in the Finnish population
Source: Health Qual Life Outcomes. 2019 Jul 8;17:117. doi: 10.1186/s12955-019-1187-9 (PMC6615142; doi:10.1186/s12955-019-1187-9)
Supplement: Supplementary file 2 — Five most frequently reported EQ-5D-5 L health states in persons with NI-T2D and non-diabetics. Five most frequently reported EQ-5D-5 L health states in respondents with NI-T2D and non-diabetics. The dimensions are in order: mobility, self-care, usual activities, pain/discomfort and anxiety/depression. (DOCX 14 kb) [file 12955_2019_1187_MOESM2_ESM.docx]

Additional file 2. Five most frequently reported EQ-5D-5L health states in persons with NI-T2D and non-diabetics. The dimensions are in order: mobility, self-care, usual activities, pain/discomfort and anxiety/depression.

| **No diabetes (n=4856)** | | | | **NI-T2D (n= 449)** | | | |
| --- | --- | --- | --- | --- | --- | --- | --- |
| Health state | Frequency | Percent | Cumulative percentage | Health state | Frequency | Percent | Cumulative percentage |
| 11111 | 1 390 | 29.3 | 29.3 | 11121 | 75 | 17.4 | 17.4 |
| 11121 | 1 245 | 26.3 | 55.6 | 11111 | 68 | 15.8 | 33.2 |
| 11122 | 313 | 6.6 | 62.2 | 21121 | 55 | 12.8 | 45.9 |
| 21121 | 292 | 6.2 | 68.4 | 21221 | 16 | 3.7 | 49.7 |
| 11112 | 150 | 3.2 | 71.5 | 11122 | 14 | 3.2 | 52.9 |
