# Supplementary material for: Impact of type 2 diabetes treated with non-insulin medication and number of diabetes-coexisting diseases on EQ-5D-5 L index scores in the Finnish population
Source: Health Qual Life Outcomes. 2019 Jul 8;17:117. doi: 10.1186/s12955-019-1187-9 (PMC6615142; doi:10.1186/s12955-019-1187-9)
Supplement: Supplementary file 3 — Characteristics of respondents who attended the postal survey and the internet survey. An overview of the demographics and the disease burden of the postal survey respondents and internet survey respondents. (DOCX 17 kb) [file 12955_2019_1187_MOESM3_ESM.docx]

Additional file 3. Characteristics of respondents who responded to the postal survey and the internet survey.

|  | **Variable** | **Population**  **(n=5 305)**  **n (%)** | **Postal survey**  **(n=3 109)**  **n (%)** | **Internet survey**  **(n=2 196)**  **n (%)** |
| --- | --- | --- | --- | --- |
|  | NI-T2D* | 449 (8.5) | 289 (9.2) | 160 (7.2) |
|  | Male* | 2 349 (44.4) | 1 279 (41.1) | 1 070 (48.7) |
|  | EQ-5D-5L index score ± SD* | 0.90 ± 0.13 | 0.89 ± 0.13 | 0.91 ± 0.12 |
|  | Age (year)* | 53.4 ± 16.2 | 55.2 ± 16.6 | 51.0 ± 15.3 |
|  | Range | 18–80 | 18–80 | 18–79 |
| Diabetes-coexisting diseases | Hypertension* | 1 564 (29.5) | 1 008 (32.4) | 556 (25.3) |
|  | Hypercholesterolemia * | 1 078 (20.3) | 699 (22.4) | 379 (17.2) |
|  | Musculoskeletal disorders* | 838 (15.8) | 517 (16.6) | 321 (14.6) |
|  | Heart problems* | 581 (11.0) | 382 (12.2) | 199 (9.0) |
|  | Sleep problems* | 383 (7.2) | 252 (8.1) | 131 (5.9) |
|  | GI disorders | 330 (6.2) | 196 (6.3) | 134 (6.1) |
|  | Kidney disease | 10 (0.2) | 7 (0.2) | 3 (0.1) |
| Number of diabetes-coexisting diseases* | 0 | 2 682 (50.5) | 1 490 (47.9) | 1 192 (54.2) |
|  | 1 | 1 281 (24.1) | 740 (23.8) | 541 (24.6) |
|  | 2 | 734 (13.8) | 470 (15.1) | 264 (12.0) |
|  | 3 | 434 (8.1) | 284 (9.1) | 150 (6.8) |
|  | 4 or more | 174 (3.2) | 125 (4.0) | 49 (2.2) |
| Marital status* | Married | 3 197 (60.4) | 1 759 (56.5) | 1 438 (65.4) |
|  | Unmarried | 1 107 (20.9) | 652 (20.9) | 455 (20.7) |
|  | Divorced or separated | 643 (12.2) | 422 (13.5) | 221 (10.0) |
|  | Widowed | 342 (6.5) | 260 (8.3) | 82 (3.7) |
| Level of education* | Elementary school | 779 (14.8) | 648 (20.8) | 131 (5.9) |
|  | High school | 415 (7.9) | 197 (6.3) | 218 (9.9) |
|  | Vocational school | 1 979 (37.6) | 1 205 (38.7) | 774 (35.2) |
|  | College | 851 (16.2) | 391 (12.5) | 460 (20.9) |
|  | University | 1 120 (21.3) | 537 (17.2) | 583 (26.5) |
|  | Other | 124 (2.4) | 94 (3.0) | 30 (1.3) |
| Household income* | Less than 1 000 € | 538 (10.3) | 362 (11.6) | 176 (8.0) |
|  | 1 001 – 2 000 € | 1 292 (24.8) | 883 (28.4) | 409 (18.6) |
|  | 2 001 – 3 000 € | 1 219 (23.4) | 727 (23.3) | 492 (22.4) |
|  | 3 001 – 4 000 € | 1 053 (20.2) | 534 (17.1) | 519 (23.6) |
|  | 4 001 – 5 000 € | 619 (11.9) | 276 (8.8) | 343 (15.6) |
|  | 5 001 – 8 000 € | 403 (7.7) | 200 (6.4) | 203 (9.2) |
|  | Over 8 000 € | 91 (1.8) | 37 (1.1) | 54 (2.4) |
| Employment* | Full time work | 2 094 (39.6) | 1 079 (34.7) | 1 015 (46.2) |
|  | Part time work or retirement | 346 (6.6) | 200 (6.4) | 146 (6.6) |
|  | Unemployed or laid off | 374 (7.1) | 203 (6.5) | 171 (7.7) |
|  | Retired | 1 942 (36.8) | 1 304 (41.9) | 638 (29.0) |
|  | Other | 526 (10.0) | 298 (9.5) | 226 (10.2) |

*p<.05 when comparing postal survey and internet survey respondents
