# Supplementary material for: Impact of type 2 diabetes treated with non-insulin medication and number of diabetes-coexisting diseases on EQ-5D-5 L index scores in the Finnish population
Source: Health Qual Life Outcomes. 2019 Jul 8;17:117. doi: 10.1186/s12955-019-1187-9 (PMC6615142; doi:10.1186/s12955-019-1187-9)
Supplement: Supplementary file 4 — A Marginal effects estimated with two-part model for the association between NI-T2D and EQ-5D-5 L disutility score (i.e., 1 – EQ-5D-5 L index score) (N = 4998). The marginal effects of the two-part model used in calculating EQ-5D-5 L index scores. 4 B Marginal effects estimated with two-part model for the association between NI-T2D and crosswalk EQ-5D-3 L disutility score (i.e., 1 – crosswalk EQ-5D-3 L index score) (N = 4998). The marginal effects of the two-part model used in calculating EQ-5D-5 L/3 L Crosswalk index scores. (ZIP 34 kb) [file 12955_2019_1187_MOESM4_ESM.zip › Additional file 4A.docx]

Additional file 4A. Marginal effects estimated with two-part model for the association between NI-T2D and EQ-5D-5L disutility score (i.e., 1 – EQ-5D-5L index score) (N=4 998).

|  |  |  | Disutility (EQ-5D-5L) | Std.Err. |  | z | P>\|z\| | 95 % Lower | 95 % upper |
| --- | --- | --- | --- | --- | --- | --- | --- | --- | --- |
| **Age** |  |  | <0.001 | <0.001 |  | -2.11 | 0.035 | <0.001 | <0.001 |
| **Gender** |  |  | -0.006 | 0.003 |  | -1.97 | 0.049 | -0.012 | <0.001 |
| **Income** |  |  |  |  |  |  |  |  |  |
| Less than 1 000 € as reference group | | | |  |  |  |  |  |  |
| 1 001 – 2 000 € | |  | -0.028 | 0.007 |  | -3.63 | <0.001 | -0.044 | -0.013 |
| 2 001 – 3 000 € | |  | -0.047 | 0.008 |  | -5.54 | <0.001 | -0.063 | -0.030 |
| 3 001 – 4 000 € | |  | -0.060 | 0.008 |  | -6.77 | <0.001 | -0.077 | -0.042 |
| 4 001 – 5 000 € | |  | -0.052 | 0.009 |  | -5.38 | <0.001 | -0.071 | -0.033 |
| 5 001 – 8 000 € | |  | -0.065 | 0.009 |  | -6.62 | <0.001 | -0.085 | -0.046 |
| Over 8 000 € | |  | -0.076 | 0.012 |  | -6.14 | <0.001 | -0.100 | -0.051 |
| **Education** | |  |  |  |  |  |  |  |  |
| Elementary school as reference group | | | |  |  |  |  |  |  |
| High school | |  | -0.006 | 0.007 |  | -0.87 | 0.385 | -0.021 | 0.008 |
| Vocational school | |  | 0.001 | 0.005 |  | 0.22 | 0.827 | -0.008 | 0.011 |
| College |  |  | -0.006 | 0.006 |  | -1.00 | 0.319 | -0.017 | 0.005 |
| University | |  | -0.012 | 0.005 |  | -2.11 | 0.035 | -0.023 | <0.001 |
| Other |  |  | 0.008 | 0.012 |  | 0.74 | 0.461 | -0.014 | 0.032 |
| **Occupation** | |  |  |  |  |  |  |  |  |
| Full time work as reference group | | | |  |  |  |  |  |  |
| Part-time work | |  | 0.010 | 0.007 |  | 1.44 | 0.150 | -0.003 | 0.023 |
| Part working, part retired | | | 0.029 | 0.011 |  | 2.58 | 0.010 | 0.007 | 0.051 |
| Unemployed | |  | 0.030 | 0.006 |  | 4.44 | <0.001 | 0.016 | 0.043 |
| Retired |  |  | 0.043 | 0.005 |  | 7.93 | <0.001 | 0.032 | 0.053 |
| Maternal leave | |  | -0.020 | 0.009 |  | -2.23 | 0.026 | -0.038 | -0.002 |
| Studying |  |  | -0.006 | 0.006 |  | -1.07 | 0.285 | -0.019 | 0.005 |
| Other |  |  | 0.126 | 0.017 |  | 7.43 | <0.001 | 0.093 | 0.160 |
| **Social status** | |  |  |  |  |  |  |  |  |
| Married as reference group | | |  |  |  |  |  |  |  |
| Unmarried | |  | -0.007 | 0.004 |  | -1.53 | 0.126 | -0.016 | 0.002 |
| Divorced |  |  | 0.002 | 0.005 |  | 0.44 | 0.660 | -0.008 | 0.014 |
| Widowed | |  | -0.019 | 0.006 |  | -2.96 | 0.003 | -0.031 | -0.006 |
| **Survey type** | |  |  |  |  |  |  |  |  |
| Postal survey as reference group | | | |  |  |  |  |  |  |
| Internet survey | | | -0.003 | 0.003 |  | -1.12 | 0.263 | -0.010 | 0.002 |
| **NI-T2D** | |  | 0.036 | 0.006 |  | 5.34 | <0.001 | 0.023 | 0.050 |

Results were also adjusted for residential area
