# Supplementary material for: Impact of type 2 diabetes treated with non-insulin medication and number of diabetes-coexisting diseases on EQ-5D-5 L index scores in the Finnish population
Source: Health Qual Life Outcomes. 2019 Jul 8;17:117. doi: 10.1186/s12955-019-1187-9 (PMC6615142; doi:10.1186/s12955-019-1187-9)
Supplement: Supplementary file 4 — A Marginal effects estimated with two-part model for the association between NI-T2D and EQ-5D-5 L disutility score (i.e., 1 – EQ-5D-5 L index score) (N = 4998). The marginal effects of the two-part model used in calculating EQ-5D-5 L index scores. 4 B Marginal effects estimated with two-part model for the association between NI-T2D and crosswalk EQ-5D-3 L disutility score (i.e., 1 – crosswalk EQ-5D-3 L index score) (N = 4998). The marginal effects of the two-part model used in calculating EQ-5D-5 L/3 L Crosswalk index scores. (ZIP 34 kb) [file 12955_2019_1187_MOESM4_ESM.zip › Additional file 4B.docx]

Additional file 4B Marginal effects estimated with two-part model for the association between NI-T2D and EQ-5D-3L crosswalk disutility score (i.e., 1 – EQ-5D-3L crosswalk index score) (N=4 998).

|  |  | Disutility (EQ-5D-3L) | | Std.Err | |  | z | P>\|z\| | 95 % lower | 95 % upper |
| --- | --- | --- | --- | --- | --- | --- | --- | --- | --- | --- |
| **Age** |  | <0.001 |  | <0.001 |  |  | 0.06 | 0.952 | <0.001 | <0.001 |
| **Gender** |  | -0.006 |  | 0.003 |  |  | -2.94 | 0.003 | -0.020 | -0.004 |
| **Income** |  |  |  |  |  |  |  |  |  |  |
| Less than 1 000 € as reference group | | |  |  |  |  |  |  |  |  |
| 1 001 – 2 000 € | | -0.029 |  | 0.007 |  |  | -3.78 | <0.001 | -0.051 | -0.016 |
| 2 001 – 3 000 € | | -0.047 |  | 0.008 |  |  | -5.19 | <0.001 | -0.069 | -0.031 |
| 3 001 – 4 000 € | | -0.060 |  | 0.009 |  |  | -6.51 | <0.001 | -0.088 | -0.047 |
| 4 001 – 5 000 € | | -0.053 |  | 0.010 |  |  | -4.92 | <0.001 | -0.079 | -0.034 |
| 5 001 – 8 000 € | | -0.066 |  | 0.010 |  |  | -5.71 | <0.001 | -0.093 | -0.045 |
| Over 8 000 € | | -0.076 |  | 0.012 |  |  | -5.04 | <0.001 | -0.119 | -0.052 |
| **Education** | |  |  |  |  |  |  |  |  |  |
| Elementary school as reference group | | | |  |  |  |  |  |  |  |
| High school | | -0.006 |  | 0.007 |  |  | -1.71 | 0.088 | -0.035 | 0.002 |
| Vocational school | | 0.001 |  | 0.005 |  |  | -0.34 | 0.734 | -0.015 | 0.010 |
| College |  | -0.006 |  | 0.006 |  |  | -1.94 | 0.053 | -0.031 | <0.001 |
| University | | -0.012 |  | 0.006 |  |  | -3.58 | <0.001 | -0.041 | -0.012 |
| Other |  | 0.009 |  | 0.012 |  |  | -0.04 | 0.971 | -0.030 | 0.029 |
| **Occupation** | |  |  |  |  |  |  |  |  |  |
| Full time work as reference group | | | |  |  |  |  |  |  |  |
| Part-time work | | 0.010 |  | 0.007 |  |  | 1.15 | 0.251 | -0.007 | 0.029 |
| Part working, part retired | | 0.029 |  | 0.011 |  |  | 2.45 | 0.014 | 0.007 | 0.063 |
| Unemployed | | 0.030 |  | 0.007 |  |  | 4.08 | <0.001 | 0.018 | 0.052 |
| Retired |  | 0.043 |  | 0.005 |  |  | 7.06 | <0.001 | 0.035 | 0.063 |
| Maternal leave | | -0.020 |  | 0.009 |  |  | -2.48 | 0.013 | -0.062 | -0.007 |
| Studying |  | -0.007 |  | 0.006 |  |  | -1.78 | 0.075 | -0.034 | 0.001 |
| Other |  | 0.127 |  | 0.017 |  |  | 8.42 | <0.001 | 0.111 | 0.179 |
| **Social status** | |  |  |  |  |  |  |  |  |  |
| Married as reference group | | |  |  |  |  |  |  |  |  |
| Unmarried | | -0.007 |  | 0.005 |  |  | -1.16 | 0.248 | -0.019 | 0.005 |
| Divorced |  | 0.003 |  | 0.006 |  |  | 0.21 | 0.837 | -0.013 | 0.016 |
| Widowed | | -0.019 |  | 0.006 |  |  | -2.78 | 0.005 | -0.043 | -0.007 |
| **Survey type** | |  |  |  |  |  |  |  |  |  |
| Postal survey as reference group | | | |  |  |  |  |  |  |  |
| Internet survey | | -0.002 |  | 0.004 |  |  | -0.55 | 0.584 | -0.01 | 0.005 |
| **NI-T2D** | | 0.047 |  | 0.008 |  |  | 5.68 | <0.001 | 0.030 | 0.062 |

Results were also adjusted for residential area
