# Supplementary material for: Impact of type 2 diabetes treated with non-insulin medication and number of diabetes-coexisting diseases on EQ-5D-5 L index scores in the Finnish population
Source: Health Qual Life Outcomes. 2019 Jul 8;17:117. doi: 10.1186/s12955-019-1187-9 (PMC6615142; doi:10.1186/s12955-019-1187-9)
Supplement: Supplementary file 5 — EQ-5D-5 L disutility scores of respondents with NI-T2D when compared to non-diabetics with no coexisting diseases. The observed differences are the marginal effect point estimates and their 95% confidence intervals. EQ-5D-5 L disutility scores of respondents with NI-T2D when compared to non-diabetics with no coexisting diseases. Comparison done in respondents of a single age: 20, 40, 60 and 80 years and accumulated coexisting diseases (0, 1, 2, 3 or 4 ≥). (DOCX 18 kb) [file 12955_2019_1187_MOESM5_ESM.docx]

Additional file 5. EQ-5D-5L index disutility scores of respondents with NI-T2D when compared to non-diabetics with no coexisting diseases. Comparison done in respondents of a single age: 20, 40, 60 and 80 years old and with a specific number of coexisting diseases (0, 1, 2, 3 or ≥4).

| **Group** | **Age** | **Disutility (EQ-5D-5L)** | **Std. Err.** | **z** | **P>\|z\|** | **95 % lower** | **95 % upper** |
| --- | --- | --- | --- | --- | --- | --- | --- |
| **No T2D, 1 coexisting disease** | 20 | 0.081 | 0.007 | 11.49 | <0.000 | 0.067 | 0.081 |
|  | 40 | 0.063 | 0.004 | 13.69 | <0.000 | 0.054 | 0.063 |
|  | 60 | 0.049 | 0.003 | 14.77 | <0.000 | 0.042 | 0.049 |
|  | 80 | 0.038 | 0.002 | 13.79 | <0.000 | 0.033 | 0.038 |
| **No T2D, 2 coexisting diseases** | 20 | 0.138 | 0.012 | 11.46 | <0.000 | 0.114 | 0.138 |
|  | 40 | 0.107 | 0.007 | 13.79 | <0.000 | 0.092 | 0.107 |
|  | 60 | 0.083 | 0.005 | 15.15 | <0.000 | 0.072 | 0.083 |
|  | 80 | 0.065 | 0.004 | 14.31 | <0.000 | 0.056 | 0.065 |
| **No T2D, 3 coexisting diseases** | 20 | 0.150 | 0.015 | 9.82 | <0.000 | 0.120 | 0.150 |
|  | 40 | 0.117 | 0.01 | 11.29 | <0.000 | 0.097 | 0.117 |
|  | 60 | 0.091 | 0.007 | 12.07 | <0.000 | 0.076 | 0.091 |
|  | 80 | 0.071 | 0.006 | 11.64 | <0.000 | 0.059 | 0.071 |
| **No T2D, 4**≥ **coexisting diseases** | 20 | 0.294 | 0.034 | 8.63 | <0.000 | 0.227 | 0.294 |
|  | 40 | 0.230 | 0.024 | 9.53 | <0.000 | 0.183 | 0.230 |
|  | 60 | 0.180 | 0.018 | 9.96 | <0.000 | 0.144 | 0.180 |
|  | 80 | 0.140 | 0.014 | 9.69 | <0.000 | 0.112 | 0.140 |
| **NI-T2D, 0 coexisting diseases** | 20 | 0.024 | 0.028 | 0.86 | 0.389 | -0.031 | 0.024 |
|  | 40 | 0.018 | 0.021 | 0.85 | 0.393 | -0.023 | 0.018 |
|  | 60 | 0.014 | 0.016 | 0.85 | 0.398 | -0.018 | 0.014 |
|  | 80 | 0.010 | 0.012 | 0.84 | 0.403 | -0.014 | 0.010 |
| **NI-T2D, 1 coexisting disease** | 20 | 0.091 | 0.019 | 4.78 | <0.000 | 0.054 | 0.091 |
|  | 40 | 0.071 | 0.014 | 4.90 | <0.000 | 0.042 | 0.071 |
|  | 60 | 0.055 | 0.011 | 4.95 | <0.000 | 0.033 | 0.055 |
|  | 80 | 0.042 | 0.008 | 4.90 | <0.000 | 0.025 | 0.042 |
| **NI-T2D, 2 coexisting diseases** | 20 | 0.095 | 0.017 | 5.61 | <0.000 | 0.062 | 0.095 |
|  | 40 | 0.074 | 0.012 | 5.82 | <0.000 | 0.049 | 0.074 |
|  | 60 | 0.057 | 0.009 | 5.90 | <0.000 | 0.038 | 0.057 |
|  | 80 | 0.044 | 0.007 | 5.83 | <0.000 | 0.029 | 0.044 |
| **NI-T2D, 3 coexisting diseases** | 20 | 0.146 | 0.020 | 7.12 | <0.000 | 0.106 | 0.146 |
|  | 40 | 0.114 | 0.014 | 7.64 | <0.000 | 0.085 | 0.114 |
|  | 60 | 0.089 | 0.011 | 7.87 | <0.000 | 0.067 | 0.089 |
|  | 80 | 0.070 | 0.009 | 7.73 | <0.000 | 0.052 | 0.070 |
| **NI-T2D, 4**≥ **coexisting diseases** | 20 | 0.334 | 0.047 | 7.04 | <0.000 | 0.241 | 0.334 |
|  | 40 | 0.261 | 0.034 | 7.47 | <0.000 | 0.192 | 0.261 |
|  | 60 | 0.203 | 0.026 | 7.64 | <0.000 | 0.151 | 0.203 |
|  | 80 | 0.158 | 0.021 | 7.50 | <0.000 | 0.117 | 0.158 |

Non-diabetics with no coexisting diseases as reference group
